# Reply to Brian and Walker-Hale: Support for the island rule does not hide morphological disparity in insular plants

**DOI:** 10.1073/pnas.1917767116

**Published:** 2019-11-26

**Authors:** M. Biddick, K. C. Burns

**Affiliations:** ^a^School of Biological Sciences, Victoria University of Wellington, Wellington 6140, New Zealand

We would like to thank Brian and Walker-Hale (1) for their insightful comments on our paper (2). Their ideas prompt us to think differently and more deeply about morphological variability in island plants. However, we would like to reconcile their reservations about the variability that is inherent in morphometric data and the traditionally employed, trait-based approach of testing the island rule (e.g., refs. 3 and 4).

Using an example from our dataset, Brian and Walker-Hale first describe how variable within-species morphology might be: “*Veronica elliptica*…leaf area…shows a 5-fold difference between 2 different sources.” Closer inspection reveals that this difference does not represent within-species morphological variation, but rather different measurement types, which we describe in the methods (maximum leaf length [*n* = 30] versus leaf area [*n* = 104]). Hypothetically, this could have influenced our results, yet restricting the analysis to leaf area measurements only, or including the two types of measurement as a random effect, yields identical conclusions to our original findings (*P* = 0.009 and *P* = 0.004, respectively).

Next, they describe how molecular phylogenetic work identified a single mainland species (*Coprosma repens*, Rubiaceae) as most closely related to two island species (cf. refs. 5 and 6), which exhibit dissimilar patterns of trait evolution. Unlike laws in physics (e.g., Newton’s law of universal gravitation), ecogeographic “laws” or “rules” describe statistical tendencies across large groups of species. Rensch’s, Bergmann’s, and Baker’s rules are core components of the scientific study of biogeography (7–9), yet they have numerous exceptions, each caused by a variety of potential factors. The island rule is no different. Deviations from it can result from numerous factors, including time since colonization, island climates, or the trait in question, as we show in our paper (2). Individual exceptions do not disprove ecogeographic rules. Rather, we think it is remarkable that support for the island rule in plant stature and leaf size persists despite the many known sources of variation in both (10).

Finally, they suggest that tests of the island rule could potentially hide disparate evolutionary trends among traits (11). As they mention, of the 94 comparisons for which both metrics were available, stature and leaf size did not evolve jointly in 36. However, the remaining 58 comparisons did, and formal analysis reveals that changes in stature and leaf size are in fact strongly correlated (Fig. 1; df = 92, *T* = 4.175, *r* = 0.399, *P* = 6.74e^−05^). So while we recognize that patterns of among-trait evolution can sometimes be disparate, they are more often congruent, and a statistical tendency in line with the predictions of the island rule remains.

**Fig. 1.**
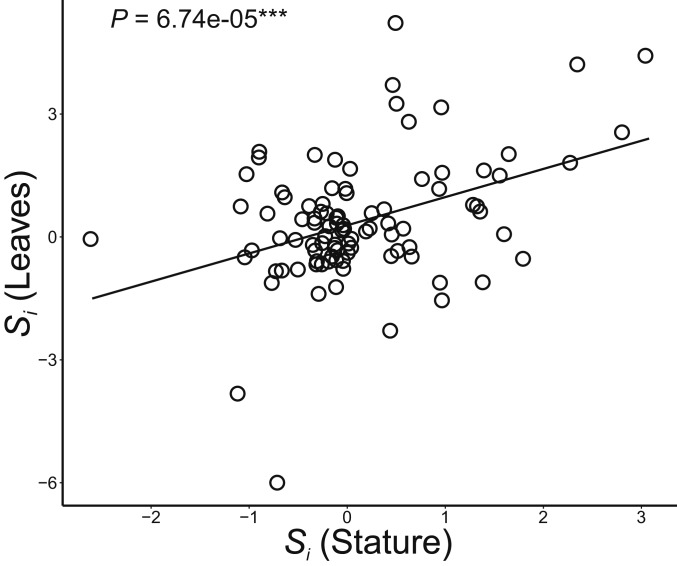
Correlation between insular size changes (S_i_ [island value/mainland value]) in plant stature and leaf size (df = 92, *T* = 4.176, *r* = 0.399, *P* = 6.74e^−05^).

As a result, it would not appear that island plants are “exploring…trait space,” as Brian and Walker-Hale suggest. It is also premature to speculate whether the evolution of island plants results from drift. So while we are grateful to Brian and Walker-Hale for their interesting and insightful ideas, plants still obey (and disobey) the island rule.

## References

[1] Brian J. I., Walker-Hale N., Focus on an island rule may hide morphological disparity in insular plants. Proc. Natl. Acad. Sci. U.S.A. 116, 24929–24930 (2019). 31772005 10.1073/pnas.1916554116 PMC6911237

[2] Biddick M., Hendriks A., Burns K. C., Plants obey (and disobey) the island rule. Proc. Natl. Acad. Sci. U.S.A. 116, 17632–17634 (2019). 31427521 10.1073/pnas.1907424116 PMC6731657

[3] Welch J. J., Testing the island rule: primates as a case study. Proc. Biol. Sci. 276, 675–682 (2009). 18957368 10.1098/rspb.2008.1180 PMC2660931

[4] Lomolino M. V., Body size evolution of insular vertebrate: Generality of the island rule. J. Biogeogr. 32, 1683–1699 (2005).

[5] Heenan P. B., Mitchell A. D., de Lange P. J., Keeling J., Paterson A. M., Late-Cenezoic origin and diversification of Chatham Islands endemic plant species revealed by analyses of DNA sequence data. N. Z. J. Bot. 48, 83–136 (2010).

[6] Cantley J. T., Swenson N. G., Markey A., Keeley S. C., Biegeographic insights on Pacific *Coprosma* (Rubiaceae) indicate two colonizations of the Hawaiian Islands. Biol. J. Linn. Soc. Lond. 174, 412–424 (2014).

[7] Kavanagh P. H., Lehnebach C. A., Shea M. J., Burns K. C., Allometry of sexual size dimorphism in dioecious plants: Do plants obey Rensch’s rule? Am. Nat. 178, 596–601 (2011). 22030729 10.1086/662175

[8] Blackburn T. M., Gaston K. J., Loder N., Geographic gradients in body size: A clarification of Bergmann’s rule. Divers. Distrib. 5, 165–174 (1999).

[9] Lomolino M. V., Sax D. F., Riddle B. R., Brown J. H., The island rule and a research agenda for studying ecogeographical patterns. J. Biogeogr. 33, 1503–1510 (2006).

[10] Wright I. J., Global climatic drivers of leaf size. Science 357, 917–921 (2017). 28860384 10.1126/science.aal4760

[11] Biddick M., Hutton I., Burns K. C., Independent evolution of allometric traits: A test of the allometric constraint hypothesis. Biol. J. Linn. Soc. Lond. 126, 203–211 (2018).

